# Stereotactic body radiotherapy vs conventionally fractionated chemoradiation in locally advanced pancreatic cancer: A multicenter case‐control study (PAULA‐1)

**DOI:** 10.1002/cam4.3330

**Published:** 2020-09-10

**Authors:** Alessandra Arcelli, Milly Buwenge, Gabriella Macchia, Federica Bertini, Alessandra Guido, Francesco Deodato, Savino Cilla, Valerio Scotti, Maria Elena Rosetto, Igor Djan, Salvatore Parisi, Gian Carlo Mattiucci, Francesco Cellini, Michele Fiore, Pierluigi Bonomo, Liliana Belgioia, Rita Marina Niespolo, Pietro Gabriele, Mariacristina Di Marco, Nicola Simoni, Renzo Mazzarotto, Alessio Giuseppe Morganti, the AIRO (Italian Association of Radiation Oncology and Clinical Oncology) Gastrointestinal Study Group

**Affiliations:** ^1^ Radiation Oncology Center Department of Experimental, Diagnostic and Specialty Medicine – DIMES University of Bologna S. Orsola‐Malpighi Hospital Bologna Italy; ^2^ Radiotherapy Unit Gemelli Molise Hospital Campobasso Italy; ^3^ Istituto di Radiologia Università Cattolica del Sacro Cuore Rome Italy; ^4^ Medical Physics Unit Gemelli Molise Hospital Campobasso Italy; ^5^ San Rossore Private Hospital Pisa Italy; ^6^ Radiotherapy Unit Belcolle Hospital Viterbo Italy; ^7^ Institute of Oncology Vojvodina Sremska Kamenica, Medical Faculty University of Novi Sad Novi Sad Serbia; ^8^ Unit of Radiation Therapy IRCCS “Casa Sollievo della Sofferenza” San Giovanni Rotondo San Giovanni Rotondo Italy; ^9^ UOC Radioterapia Oncologica Dipartimento di Diagnostica per Immagini Radioterapia Oncologica ed Ematologia Fondazione Policlinico Universitario "A. Gemelli" IRCCS Rome Italy; ^10^ Radiation Oncology Campus Bio‐Medico University Rome Italy; ^11^ Radiation Oncology Azienda Ospedaliero‐Universitaria Careggi Florence Italy; ^12^ Department of Radiotherapy Policlinico San Martino University of Genoa Genoa Italy; ^13^ Radiotherapy Unit Azienda Ospedaliera San Gerardo Monza Italy; ^14^ Radiation Therapy Candiolo Cancer Institute – FPO IRCCS Candiolo Candiolo Italy; ^15^ Oncology Unit Department of Experimental Diagnostic and Specialty Medicine – DIMES, University of Bologna S. Orsola‐Malpighi Hospital Bologna Italy; ^16^ Department of Radiotherapy Azienda Ospedaliera Universitaria Integrata Verona Italy

**Keywords:** conventionally fractionated radiotherapy, pancreatic cancer, radiation therapy, stereotactic body radiotherapy

## Abstract

Conventionally fractionated chemoradiation (CRT) or chemotherapy (CHT) are considered as standard options in locally advanced pancreatic cancer (LAPC) while stereotactic body radiotherapy (SBRT) is an emerging treatment in this setting. The aim of this study was to compare two cohorts of LAPC patients treated with SBRT ± CHT vs CRT ± CHT in terms of local control (LC), distant metastases‐free survival (DMFS), progression‐free survival (PFS), overall survival (OS), and toxicity. Eighty patients were included. Patients in the two cohorts were matched according to: age ≤/>65 years, tumor diameter (two cut‐offs: </≥3.0 and </≥3.9 cm), clinical tumor stage and clinical nodal stage, neoadjuvant CHT, and adjuvant CHT. Median prescribed total dose was 30.0 Gy (range: 18.0‐37.5) and 54.0 Gy (18.0‐63.0) in SBRT and CRT cohorts, respectively. Toxicity was evaluated by CTCAE v4.0 scale. Survival curves were calculated by Kaplan‐Meier method. For hypothesis testing an equivalence and a non‐inferiority test was calculated. No statistically significant differences in terms of acute and late toxicity, DMFS, PFS, and OS were recorded among the two cohorts. Median, 1‐, and 2‐year LC was: 16.0 months, 53.1%, and 40.5% in the CRT cohort and 22.0 months, 80.4%, and 49.8% in the SBRT cohort, respectively (*P*: .017). A statistically non‐inferiority significance was recorded in terms of OS between CRT and SBRT (*P* = .031). Patients treated with SBRT showed higher LC rate and similar OS compared to CRT. Therefore, the design of confirmatory randomized studies comparing SBRT and CRT seems justified.

## Graphical abstract

An equivalence of stereotactic body radiotherapy (SBRT) and chemoradiation (CRT) has been only suggested in some retrospective studies in pancreatic cancer (PC). This is the first matched case‐control study comparing SBRT and CRT in PC considering several endpoints. An equivalence in terms of overall survival, disease free survival, and metastasis free survival among the two treatments and an advantage of SBRT in terms of local control were recorded. Therefore, SBRT is almost comparable to standard CRT.

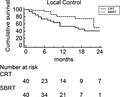

## 1 INTRODUCTION

Pancreatic cancer (PC) is a dismal disease with 8% 5‐year overall survival (OS) rate.^1^ It represents the fourth leading cause of mortality in the USA. Epidemiological studies predict that in 2030 PC will rise to second place in the same country.^2^ Moreover, only 20% of highly selected patients have a potentially resectable disease whereas 30%‐40% of patients present at diagnosis with nonmetastatic unresectable locally advanced PC (LAPC)^3^.

Chemotherapy (CHT) and/or chemoradiation (CRT) are considered as treatment options for LAPC^4^ despite conflicting results from the randomized trials that compared these two strategies.^5^, ^6^, ^7^ Particularly, median OS of LAPC patients treated with CRT plus CHT ranges from 9 to 16 months in the randomized trials published since 2000.^5^, ^6^, ^8^, ^9^

Stereotactic body radiotherapy (SBRT) is an emerging radiotherapy technique, that was pioneered in the LAPC setting by the Stanford group since 2004.^10^ The highly conformed dose distribution achievable with SBRT allows the delivery of high biologically effective doses (BED) with the potential to overcome the PC radio‐resistance and therefore improving local control (LC).^11^, ^12^, ^13^ Moreover considering the short duration, SBRT favors the sequential combination with CHT. In fact, SBRT can be completed in a few days unlike standard CRT whose duration is generally between 4‐5 weeks. Based on these potential advantages, studies comparing SBRT and CRT seem to be justified. However, only few retrospective analyses are currently available.^14^, ^15^, ^16^, ^17^

Therefore, we performed a matched case‐control study comparing two cohorts of LAPC patients treated with SBRT ± CHT or CRT ± CHT in terms of LC, progression‐free survival (PFS), distant metastases‐free survival (DMFS), and OS. The aim of this report was to present the results of this analysis.

## 2 MATERIALS AND METHODS

### 2.1 Study design

This is a multicentric, retrospective, case‐control study. On behalf of the AIRO (Italian Association of Radiation Oncology and Clinical Oncology) Gastrointestinal Study Group, we collected clinical data on 419 patients from 15 Italian centers. In our database, LAPC patients could have been treated with every possible combination and schedules of CHT and radiotherapy delivered with any technique.

For the purpose of this analysis we selected all LAPC patients (56) treated with SBRT from six different Italian centers. We then matched these 56 SBRT patients with the ones treated with CRT (298) according to the following criteria: age ≤/>65 years, tumor diameter (</≥3 cm, and </≥3.9 cm), clinical tumor stage (cT), clinical nodal stage (cN), administration of neoadjuvant and adjuvant CHT. Matching was performed, blinded to patient outcome, in a 1:1 ratio and when multiple patients matched, one was selected at random. At the end of this selection we obtained two cohorts of 40 patients each, treated with SBRT or CRT, respectively.

### 2.2 Endpoints

The purpose of this analysis was to compare SBRT ± CHT and CRT ± CHT in LAPC patients in terms of different outcomes: LC, DMFS, PFS, and OS. Our aim was also to test the non‐inferiority of SBRT compared to CRT.

### 2.3 Eligibility

LAPC patients without metastatic disease and not previously treated with surgery due to PC or with abdominal radiotherapy were included in this study.

### 2.4 Treatment

Details about SBRT treatment were previously described.^18^ CRT patients were planned and treated in supine position using a customized foam cradle. CT‐simulation was performed with intravenous and oral contrast. CRT was delivered using three‐dimensional conformal radiotherapy (70.0%), intensity modulated radiotherapy (IMRT) (20.0%), or volumetric modulated arc therapy (VMAT) (10.0%). The clinical target volume (CTV) was defined as the gross tumor volume plus a 1‐2 cm margin in the pancreatic parenchyma. Regional nodes were included in the CTV based on the tumor site. The planning target volume was defined as the CTV plus an anisotropic margin of 0.5‐1 cm radially and 1‐2 cm in cranial‐caudal direction in most patients. In 57% of patients, the planning target volume was defined using a 4D‐CT‐simulation. Dose specification and prescription were based on ICRU (International Commission on Radiation Units & Measurements) report 62 and 83 for three‐dimensional conformal radiotherapy and IMRT/VMAT, respectively. All patients were treated with conventionally fractionated radiotherapy (1.8‐2 Gy/fraction) plus concurrent CHT.

### 2.5 Follow‐up

The first follow‐up visit was carried out 3 weeks after the end of radiotherapy. Further evaluations were planned with 3 months intervals. Patients were monitored with standard blood tests, medical history, physical examination, and contrast enhanced CT scans of chest and abdomen.

### 2.6 Statistical analysis

Descriptive statistics included median and percentages for continuous and categorical variables, respectively. Categorical variables were compared using the Pearson's Chi‐square test. For hypothesis testing an equivalence and a non‐inferiority test was calculated. Survival curves were calculated using the Kaplan‐Meier method^19^ and compared using the log‐rank test.^20^ A multivariable Cox model^21^ was built to test if some clinical and pathological factors could influence outcomes. All tests were two‐sided and a *P* < .05 was considered significant. All endpoints were calculated from the date of radiotherapy start. Statistical analysis was performed with IBM SPSS Version 22.0 (IBM Corp) and Statgraphics software systems (full system 5.25 version 4.0‐ Graphics system by Statistical Graphics Corporation Ed, United States, 1989). Toxicity was scored using the CTCAE v. 4.0 scale.

### 2.7 Ethical issues

All enrolled patients signed a written informed consent. The study (PAULA‐1: Pooled Analysis in Unresectable Locally Advanced pancreatic cancer) was approved by our institutional review board (201/2015/O/OssN).

## 3 RESULTS

The characteristics of patients and treatment in the two cohorts are shown in Table 1. Median follow‐up was 15 months (range: 3‐70). Median total dose, median dose per fraction, and median total BED_α/β10Gy_, were 30.0 Gy (range: 18.0‐37.5), 6.0 Gy (range: 5.0‐10.0), and 48.0 Gy (range: 28.8‐65.6) in the SBRT cohort while the corresponding values were 50.4 Gy (range: 18.0‐63.0), 1.8 Gy (range: 1.8‐2.1), and 59.4 Gy (range: 21.2‐76.2) in the CRT cohort.

**TABLE 1:** Comparison between the two cohorts of patients treated with chemoradiation and SBRT

Variable	Value	CRT	SBRT	*P*
Age (y)	Median (range)	67 (36‐89)	67 (36‐83)	
	≤65	17 (42.5)	17 (42.5)	.589
	>65	23 (57.5)	23 (57.5)	
Gender	Male	24 (60.0)	27 (67.5)	.321
	Female	16 (40.0)	13(32.5)	
ECOG PS	0	22 (55.0)	20 (50.0)	.493
	1	16 (40.0)	15 (37.5)	
	2	2 (5.0)	5 (12.5)	
Tumor site	Head	28 (70.0)	24 (60.0)	.638
	Body	10 (25.0)	13 (32.5)	
	Tail	2 (5.0)	3 (7.5)	
Tumor diameter (cm)	Median (range)	4.0 (1.2‐8.7)	4.0 (2.0‐7.0)	
	<3.0	5 (12.5)	5 (12.5)	.631
	≥3.0 and <3.9	18 (32.5)	18 (32.5)	
	≥3.9	22 (55.0)	22 (55.0)	
cT stage	3	11 (27.5)	11 (27.5)	.599
	4	29 (72.5)	29 (72.5)	
cN stage	0	22 (55.0)	22 (55.0)	.589
	1	18 (45.0)	18 (45.0)	
Biliary stent	No	15 (37.5)	19 (47.5)	.078
	Yes	23 (57.5)	13 (32.5)	
	Unknown	2 (5.0)	8 (20.0)	
Neoadjuvant chemotherapy	No	16 (40.0)	16 (40.0)	.590
	Yes	24 (60.0)	24 (60.0)	
Neoadjuvant chemotherapy regimen	Gemcitabine	8 (33.3)	3 (12.5)	.002*
	Folfox	1 (4.2)	1 (4.2)	
	Folfirinox	2 (8.3)	6 (25.0)	
	Gemcitabine + Nab‐placlitaxel	0 (0.0)	9 (37.5)	
	Gemcitabine + Oxaliplatinum	13 (54.2)	5 (20.8)	
Adjuvant chemotherapy	No	31 (77.5)	31 (77.5)	.605
	Yes	9 (22.5)	9 (22.5)	
Adjuvant chemotherapy regimen	Gemcitabine	7 (77.8)	2 (22.2)	.073
	5‐Fluorouracil	0 (0.0)	1 (11.1)	
	Folfirinox	1 (11.1)	4 (44.4)	
	Gemcitabine + Nab‐placlitaxel	0 (0.0)	2 (22.2)	
	Gemcitabine + Oxaliplatinum	1 (11.1)	0 (0.0)	
Acute gastrointestinal toxicity	0	24 (60.0)	31 (77.5)	.175
	1	12 (30.0)	8 (20.0)	
	2	4 (10.0)	1 (2.5)	
Late gastrointestinal toxicity	0	35 (92.1)	39 (97.5)	.244
	1	1 (2.6)	0 (0.0)	
	2	2 (5.3)	0 (0.0)	
	3	0 (0.0)	1 (2.5)	

Abbreviation: CHT, chemotherapy; CRT, chemoradiotherapy; ECOG PS, Eastern Cooperative Oncology Group performance status; SBRT, stereotactic body radiotherapy.

* Significant *P* value.

The prescribed concurrent CHT regimens were gemcitabine‐ (80.0%) or capecitabine‐based (20.0%). In both cohorts, 60.0% and 22.5% patients underwent neoadjuvant and adjuvant CHT, respectively. Details on the CHT regimens used before and after radiotherapy in the two cohorts are shown in Table 1.

There were no statistically significant differences neither in terms of acute (*P* = .175) nor late gastrointestinal toxicity (*P* = .244) comparing LAPC patients treated with SBRT or CRT, respectively. Only one case (2.5%) of gastrointestinal bleeding was recorded 9 months after SBRT.

**FIGURE 1:**
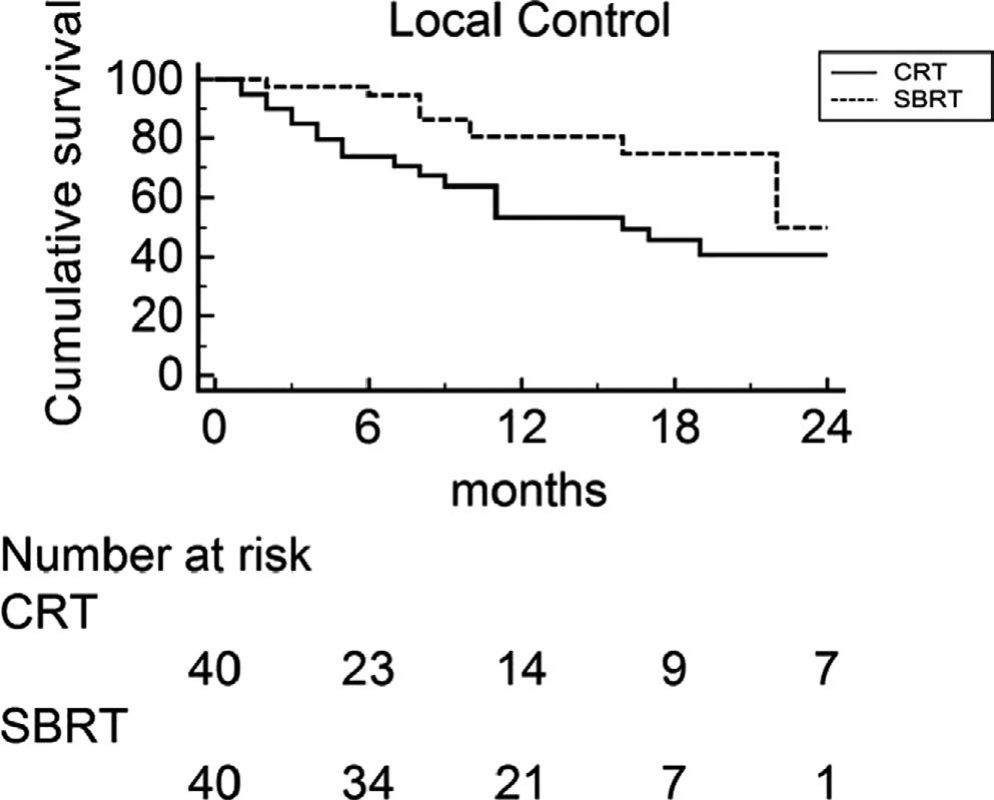
Local control of the two cohorts of patients treated with external beam chemoradiation (CRT) vs stereotactic body radiotherapy (SBRT)

At univariate analysis, there were no differences between SBRT and CRT treatment in terms of OS (*P* = .470), PFS (*P* = .749) and DMFS (*P* = .610) (Table 2). Patients treated with SBRT had a statistically significant LC improvement (Figure 1) compared to those treated with CRT (median LC: 22 months vs 16 months, respectively; *P* = .017).

**TABLE 2:** Characteristics and main findings of studies comparing SBRT +/− CHT vs CRT +/− CHT in locally advanced pancreatic cancer

Author, year	Study design	Patients	No patients of the compared treatment	Main findings
Lin, 2015^14^	Retrospective	41	20 SBRT +/− cCHT vs 21 IMRT +/− cCHT	Median, 1‐y OS: 20.0 mo vs 13.0 mo, 80.0% vs 70.7% (*P* = .127) Median, 1‐y LC: 17.5 mo vs 10.0 mo, 70% vs 37.0% (*P* = .004)
Park, 2017^16^	Retrospective unmatched cohort	270	44 SBRT +/− iCHT vs 226 IMRT +/− iCHT +/− cCHT	1‐ and 2‐y OS: 56.2%, 25.7% vs 59.6%, 27.2% (*P* = .75) 1‐ and 2‐y LF: 34.4%, 48.7% vs 30.2%, 45.5% (*P* = .51) 1‐y DF: 61.7% vs 52.4% (*P* = .25) 1‐y DF + LF: 71.5% vs 63.5% (*P* = .18) G2‐G3 GI acute toxicity: 7% vs 24% (*P* = .008); 0% vs 2% (*P* = 1.00) Resection rate: 7% vs 17% (*P* = .11)
de Geus, 2017^15^	Registry study (NCDB) unmatched cohort	14 331	5464 CHT vs 6418 CRT vs 322 SBRT + CHT vs 2127 IMRT + cCHT	Median OS: 9.9 mo vs 10.9 mo vs 13.9 mo vs 12.0 mo, (*P* < .001)
	matched cohort^a^	644	322 SBRT + CHT vs 322 CHT 322 SBRT + multiagent CHT vs 322 multiagent CHT 322 SBRT + CHT vs 322 CRT 322 SBRT + CHT vs 322 IMRT + cCHT	Median OS: 13.9 mo vs 10.2 mo, (*P* < .001) Median OS: 14.8 mo vs 12.9 mo (*P* = .095) Median OS: 13.9 mo vs 11.6 mo, (*P* = .018) Median OS: 13.9 mo vs 12.2 mo, (*P* = .049)
Zhong, 2017^17^	Registry study (NCDB) unmatched cohort	8450	631 SBRT vs 7819 CRT	Resection rate: 10.8% vs 9.2% (*P* = .410) Negative resection margin: 92% vs 84% (*P* = .062) 2‐y OS: 20.3% vs 16.3% (*P* < .001)
	matched cohort^b^	988	494 SBRT vs 494 CRT	Median OS: 13.9 mo vs 11.6 mo, (*P* < .001) 2‐y OS: 21.7% vs 16.5% (*P* = .001)
Chapman, 2018^22^	Retrospective unmatched cohort	29	22 SBRT + iCHT vs 7 IMRT + iCHT	Median PFS: 8.6 mo vs 12.5 mo (*P* = .349) Median OS: 19.7 mo vs 21.1 mo (*P* = .966)
Present study	Retrospective matched cohort^c^	80	40 SBRT +/− CHT vs 40 CRT +/− CHT	Median, 1‐ and 2‐y OS: 16.0 mo vs 21.0 mo, 79.8% vs 73.8%, 14.7% vs 40.1% (*P* = .470) Median, 1‐ and 2‐y LC: 22.0 mo vs 16.0 mo, 80.4% vs 53.1%, 49.8% vs 40.5% (*P* = .017) Median, 1‐y and DMFS: 16.0 mo vs 12.0 mo, 64.5% vs 49.3%, 20.3% vs 41.7% (*P* = .610) Median, 1‐ and 2‐y PFS: 14.0 mo vs 12.0 mo, 59.1% vs 49.2, 59.1% vs 32.4% (*P* = .749) GI acute toxicity: G1: 20.0% vs 30.0%; G2: 2.5% vs 10.0% (*P* = .175) GI late toxicity: G1: 0.0% vs 2.6%; G2: 0.0% vs 5.3%; G3 2.5% vs 0.0% (*P* = .244)

Abbreviations: cCHT, concomitant chemotherapy; CHT, chemotherapy; CRT, chemoradiotherapy; DF, distant failure; DMFS, distant metastases‐free survival; G, grade; GI, gastrointestinal; iCHT, induction chemotherapy; IMRT, intensity‐modulated radiation therapy; LC, local control; LF, local failure; NCDB, National Cancer Database; OS, overall survival; PFS, progression‐free survival; SBRT, stereotactic body radiotherapy.

^a^ By: age, sex, race, comorbidity, insurance, type of treatment center, tumor location (head or body), clinical stage.

^b^ By: age, Charlson score, AJCC clinical T and N staging, median tumor size, CT use, year of diagnosis, receipt of surgery.

^c^ By: age, AJCC clinical T and N staging, tumor diameter, neoadjuvant CT use, adjuvant CT use.

**FIGURE 2:**
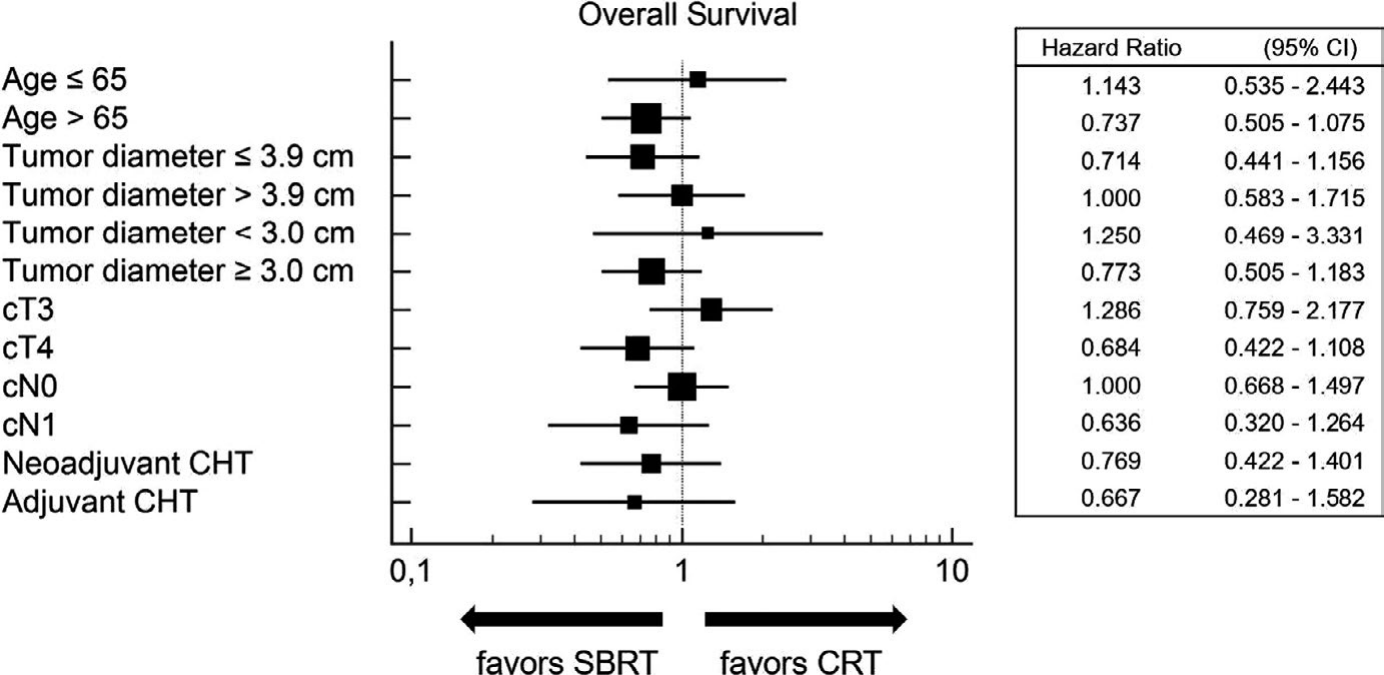
Multivariate subgroup analyses of the effects of patient characteristics on overall survival, comparing patients treated with external beam chemoradiation (CRT) vs stereotactic body radiotherapy (SBRT)

**FIGURE 3:**
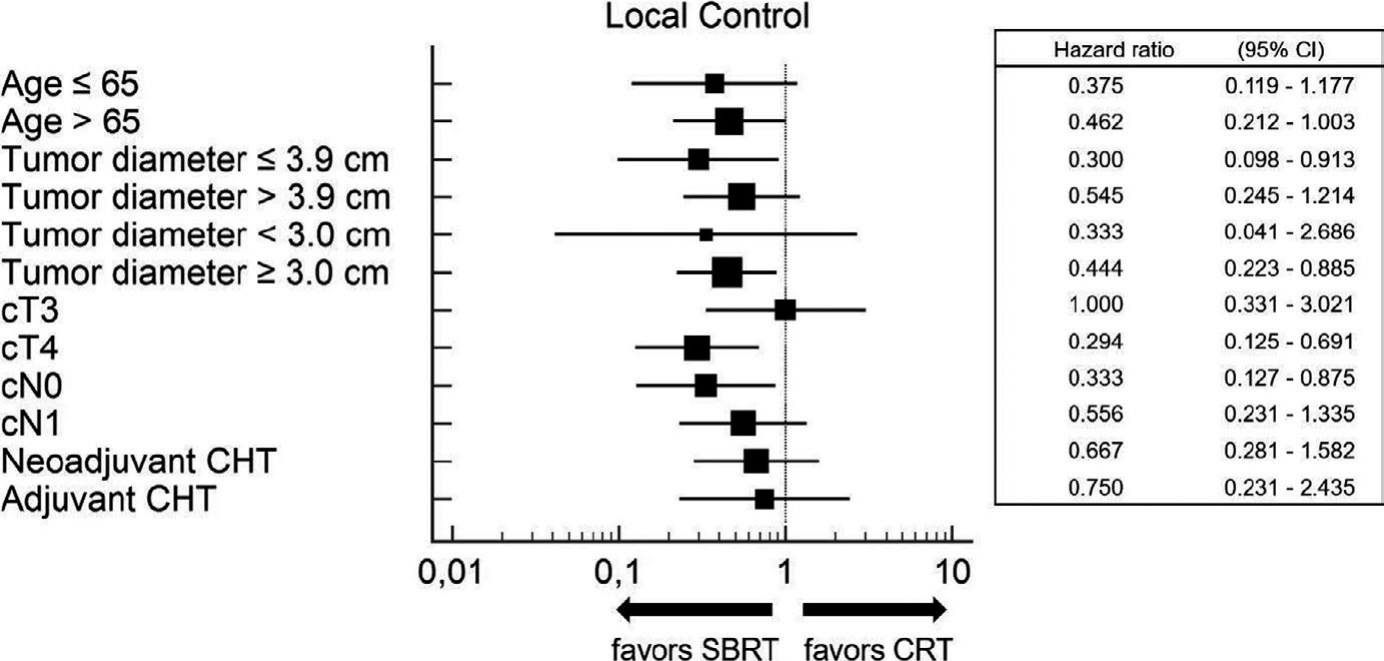
Multivariate subgroup analyses of the effects of patient characteristics on local control, comparing patients treated with external beam chemoradiation (CRT) vs stereotactic body radiotherapy (SBRT)

Figures 2 and 3 represent the multivariate sub‐group analyses of the effects of patients' demographics, disease characteristics, and treatment details of both treatment impact on OS and LC. SBRT was associated with improved LC in the subsets of patients with tumor diameter ≤ 3.9 cm, tumor diameter ≥ 3.0 cm, cT4 and cN0 stage, while in no subset was there any advantage in terms of OS from the two therapeutic modalities.

Finally, a statistically significant non‐inferiority in terms of OS was demonstrated between patients treated with SBRT and CRT (*P* = .031).

## 4 DISCUSSION

To the best of our knowledge, this is the first matched case‐control study in LAPC patients comparing conventionally fractionated CRT and SBRT in terms of different clinical outcomes. No differences in terms of OS, PFS, and DMFS were recorded while an improved LC in the SBRT cohort was registered.

Previously, some nonmatched studies^16^, ^22^ directly compared these two treatments reporting no significant differences in terms of outcomes. However, de Geus et al^15^ and Zhong et al^17^ compared matched cohorts treated with SBRT and CRT reporting improved median OS in the SBRT patients' group (Table 2). In fact, de Geus et al,^15^ in a registry study from the National Cancer Data Base on LAPC, reported higher median OS after SBRT plus CHT compared to CHT alone (*P* < .001), to standard radiotherapy plus CHT (*P* = .018), and to IMRT plus CHT (*P* = .049). In another analysis also from the National Cancer Data Base,^17^ a higher 2‐year OS rate was recorded in the SBRT ± CHT cohort, compared to conventionally fractionated radiotherapy ± CHT (*P* < .001) (Table 2). Similarly, in the meta‐analysis of Tchelebi et al^23^, including nine studies on SBRT and 11 studies on CRT in LAPC (1147 patients), an improved 2‐year OS in SBRT patients was reported (26.9% vs 13.7%, respectively; *P* = .004).

Unlike the studies mentioned above,^15^, ^17^, ^23^ our study did not show significant differences between SBRT and CRT in terms of OS. This difference could be due to the relatively small sample size of our series and to the relatively low BED_α/β10Gy_ delivered in our SBRT cohort. In fact, median BED_α/β10Gy_ was significantly lower in the latter compared to the CRT cohort (48.0 Gy vs 59.4 Gy, respectively; *P* < .001). The significant correlation recently reported by our group among BED_α/β10Gy_ ≥ 48 Gy and improved OS in SBRT of LAPC^18^ seems to confirm that the lack of improved OS in our SBRT cohort could depend on the relatively low BED_α/β10Gy_.

As mentioned above, the most interesting result of our analysis is the higher LC rate in patients undergoing SBRT compared to CRT, despite the lower median BED_α/β10Gy_ in the SBRT cohort. This difference could be explained by the extremely shorter duration of SBRT compared to CRT which could prevent tumor repopulation during therapy. Similarly, in their retrospective unmatched study, Lin et al^14^ reported significantly improved LC for LAPC patients treated with SBRT plus CHT compared to IMRT plus CHT. On the contrary, in their unmatched comparison, Park et al^16^ did not observe significant differences in terms of LC between SBRT ± induction CHT and IMRT ± induction CHT. These conflicting results (Table 2) justify the design of randomized studies which may clarify this topic.

More generally, the results recorded in our two cohorts are similar to the ones reported in other studies on SBRT or CRT in LAPC. In fact, 1‐year LC was 80.4% in our SBRT cohort, which is consistent with the pooled 1‐year LC (72.3%) reported in the systematic review of Petrelli et al^24^ on 1009 patients treated with SBRT in LAPC. Similarly, the median LC was 16 months in our CRT cohort, hence consistent with the median LC reported in the two arms of the SCALOP trial on conventionally fractionated CRT in LAPC (12.0 and 14.6 months).^9^ Similar analogies can be observed in terms of OS. Our result in terms of median OS in the SBRT cohort (16 months) is similar to that of the two systematic reviews of Petrelli et al^24^ (17 months) and Brunner et al^25^ (11 months). Moreover our results in terms of median OS (21.0 months) in the CRT cohort were at least not inferior to those reported in the SCALOP^9^ and LAP07^6^ randomized trials (13.4‐15.2 months). Beyond the case‐control design of our analysis, the relative analogy between the results recorded by us with those reported in literature makes the findings of our comparison further reliable.

Our analysis showed no significant differences in terms of both acute and late toxicity between SBRT and CRT. This result contrasts with those reported in other studies. Indeed, Park et al^16^ recorded significantly lower acute gastrointestinal toxicity grade ≥ 2 rates using SBRT compared to IMRT (*P* = .008). Moreover the metanalysis of Tchelebi et al^23^ showed a significantly higher grade 3‐4 acute toxicity in patients treated with standard radiotherapy compared to SBRT, while no differences between the two treatments were recorded in terms of late toxicity. The lack of difference in terms of toxicity observed in our series may be due to several factors such as the small sample size and the retrospective study design. In fact, the latter could have led to an incomplete recording of adverse events. Moreover the impact of the small sample size on the failure to detect differences in toxicity seems confirmed by the enrolment of only 40 patients in a study reporting similar adverse event rates between SBRT and IMRT (Table 2).^14^ Obviously, also this topic deserves further investigations.

As in any retrospective analysis our study has intrinsic limitations. Even if we used several matching criteria, the assignment to SBRT or CRT was not randomized. Therefore, we cannot rule out that our analysis is affected by bias. Particularly, although the percentage of patients undergoing neoadjuvant and adjuvant CHT was the same in the two cohorts, the used regimens were different among them. Furthermore, the relatively small sample size may have limited the possibility to detect significant differences, particularly in the subset analyses.

In conclusion, our comparison between SBRT and CRT suggests the equivalence in terms of most outcomes among the two techniques. Furthermore, for the first time using a case‐control methodology, an advantage of SBRT in terms of LC was recorded. This result, together with the logistical advantage of SBRT shorter duration, makes this technique an acceptable option in the treatment of LAPC in combination with CHT. Prospective trials are needed to better compare these two treatments. Moreover considering that in most cases LAPC treatment has a palliative purpose, these studies should include an accurate assessment of quality of life and symptoms control, especially in terms of pain relief. In fact, both conventional radiotherapy^26^ and SBRT^27^ are able to improve this symptom but direct comparisons of their relative effectiveness are lacking. Finally, considering that the only possibility of cure for patients with LAPC is to achieve a tumor downstaging to allow a radical surgical resection, the rate of resectability after SBRT and CRT should represent another relevant end point.

## CONFLICT OF INTEREST

No actual or potential conflicts of interest do exist regarding this paper.

## AUTHOR CONTRIBUTIONS

Conceptualization and design were performed by AGM, AA, GM, and MB. FB, AG, FD, SC, VS, MER, ID, SP, GCM, FC, MF, PB, LB, RMN, PG, NS, MDM, and RM contributed to data collection. Analysis and interpretation of data were performed by AA, AGM, MB, AG, FB, MDM, GM, RM, and FD. AA, AGM, MB, RM and AG contributed to manuscript writing, and NS, RM, FC, GCM, PG, PB, VS, LB, GM, and MDM contributed to the critical review of the manuscript. All Authors read and approved the final manuscript and gave consent to publication.

## Data Availability

The data that support the findings of this study are available from the corresponding author upon reasonable request.
